# Molecular mechanisms of root expansion in medicinal plants

**DOI:** 10.3389/fpls.2026.1830107

**Published:** 2026-07-27

**Authors:** Mengfan Deng, Yi Lv, Shilin Zhang, Duanyang Li, Siji Xie, Yang Xu, Chaolong Rao, Cheng Peng, Jihai Gao

**Affiliations:** 1 School of Medical and Life Sciences, Chengdu University of Traditional Chinese Medicine, Chengdu, China; 2 School of Pharmacy, Chengdu University of Traditional Chinese Medicine, Chengdu, China; 3 Chengdu Botanical Garden (Chengdu Park Urban Institute of Plant Science), Chengdu, China; 4 State Key Laboratory of Southwestern Chinese Medicine Resources, Pharmacy School, Chengdu University of Traditional Chinese Medicine, Chengdu, China

**Keywords:** influencing factors, medicinal plants, molecular mechanisms, root expansion, tissue structure

## Abstract

Medicinal plants with storage root expansion play essential roles in food supply, disease prevention, and therapeutic applications. Storage-root enlargement is a highly coordinated developmental process involving primary root specialization, secondary growth, and organ-specific differentiation. Recent studies have revealed that endogenous phytohormones, including auxin, cytokinin, gibberellin, abscisic acid, and jasmonic acid, regulate this process through interconnected signaling pathways that control cell division, cambial activity, and radial growth. Transcription factors such as the ARF, AUX/IAA, NAC, and PLT families function as central regulatory nodes that integrate hormonal and environmental signals. These regulatory networks modulate lignin deposition, carbohydrate metabolism, and secondary meristem activity. These coordinated processes ultimately drive structural remodeling and functional differentiation during root enlargement. Environmental factors, including temperature, CO_2_ concentration, nutrient availability, water status, and rhizosphere1830107 microbial communities, further influence these molecular pathways by reshaping hormone balance, assimilate allocation, and root developmental programs. Despite recent progress, the core regulatory modules and species-specific mechanisms underlying storage-root enlargement in medicinal plants remain poorly understood. This review synthesizes current knowledge on the hormonal, transcriptional, and environmental regulation of storage-root enlargement in medicinal plants and highlights future research directions to improve root yield, medicinal quality, and bioactive compound accumulation.

## 1 Introduction

Roots are essential plant organs that mediate water and nutrient uptake, anchorage, storage, and long-distance transport within the plant body. Historically, swollen or tuberous roots were first used as food resources before their medicinal value was recognized. For example, *Devil’s* Claw (*Harpagophytum procumbens*) develops large tuberous roots and has traditionally been used in soups to relieve gastrointestinal discomfort and arthritic symptoms (Brendler, 2021). Modern pharmacological studies have further demonstrated its anti-inflammatory, analgesic, and digestive effects, as well as its potential to alleviate arthritis-related complaints. In addition, genistein derived from this species may have potential benefits for the management of cardiovascular disorders (Gxaba and Manganyi, 2022). In traditional Chinese medicine, the root of *Pastinaca sativa* is described as pungent, sweet, and warm in nature and is used to dispel cold from the bladder and liver meridians and to eliminate wind-dampness (Kenari et al., 2021).

The use of underground plant organs, particularly hypertrophied structures such as roots and rhizomes, has been widely documented in traditional medical systems worldwide. In addition to traditional Chinese medicine, ancient medical systems in India, Arabia, and the Mediterranean developed extensive knowledge of the medicinal uses of these organs. Comparative analysis of traditional medical practices across different regions may therefore provide valuable insights into the therapeutic potential of medicinal plants. In traditional Indian medicine, the rhizome of *Curcuma longa* L. is a typical example of a hypertrophied underground organ of medicinal importance. Turmeric rhizomes are rich in curcuminoids, which are regarded as the major bioactive constituents responsible for their pharmacological activities. Traditionally, the rhizomes are dried and ground into a powder, which can be applied externally as a paste or administered orally (Prasad et al., 2021).

In the Arab region, traditional medical practices have also involved the extensive use of underground plant organs. Although *Boswellia sacra* Flueck is primarily valued for its resin rather than for underground tissues, its traditional use reflects the broader regional emphasis on plant-derived metabolites. The resin has been used as incense for air purification and healing or mixed with plaster for anti-inflammatory and wound-healing applications (Devi et al., 2021). In traditional Mediterranean medicine, the rhizome of *Glycyrrhiza glabra* L. is a typical medicinal underground organ. Licorice rhizomes are commonly dried and processed into aqueous preparations or chewable tablets for oral administration. Traditionally, licorice has been used to soothe cough-related throat irritation and to treat digestive disorders, including gastric ulcers, consistent with its well-documented antitussive and gastroprotective properties (Burr-Hersey et al., 2020; Zanetti et al., 2024).

For medicinal plants with underground storage organs, root quality and yield are major determinants of market value and cultivation profitability. Accordingly, enlarged underground organs are generally preferred in medicinal plant production. Root enlargement gives rise to diverse morphological structures, including tubers, enlarged primary roots, tuberous roots, hypocotyl–root transition organs, rhizomes, and bulbs (Zierer et al., 2021). According to the *Pharmacopoeia of the People’s Republic of China* 2020 Edition, Part I, 169 root- and rhizome-derived medicinal materials from 52 families are recorded, representing 27.44% of the 616 medicinal herbs and formulations listed. The families with the largest numbers of recorded root- or rhizome-derived species include Liliaceae (17 species), Ranunculaceae (14), Umbelliferae (12), Leguminosae (10), Asparagaceae (10), Zingiberaceae (9), and Asteraceae (8). These medicinal materials are mainly derived from roots, tubers, rhizomes, and bulbs. Specifically, 73 species from 32 families use roots or tubers as medicinal parts; 46 species from 20 families use rhizomes; 27 species from 17 families use both roots and rhizomes; and 23 species from 7 families use tubers or bulbs.

Root enlargement is a complex biological process regulated by genetic factors, environmental cues, secondary metabolism, and endogenous hormone signaling. In recent years, root biology has attracted increasing attention in plant science because of its importance for improving the yield and quality of edible and medicinal crops. Understanding the molecular mechanisms underlying root enlargement and their relationship with bioactive compound biosynthesis is essential for germplasm improvement, optimized cultivation practices, and the sustainable use of traditional medicinal plants.

## 2 Types and organizational characteristics of root expansion in medicinal plants

Root development gives rise to diverse morphological forms. During growth, medicinal rootstocks may develop into conical, cylindrical, fusiform, fibrous, or tuberous structures. Based on developmental origin and morphology, underground storage organs can be broadly classified into enlarged roots, which lack nodes and internodes, and underground stems, which possess distinct nodes and internodes.

**Figure 1:**
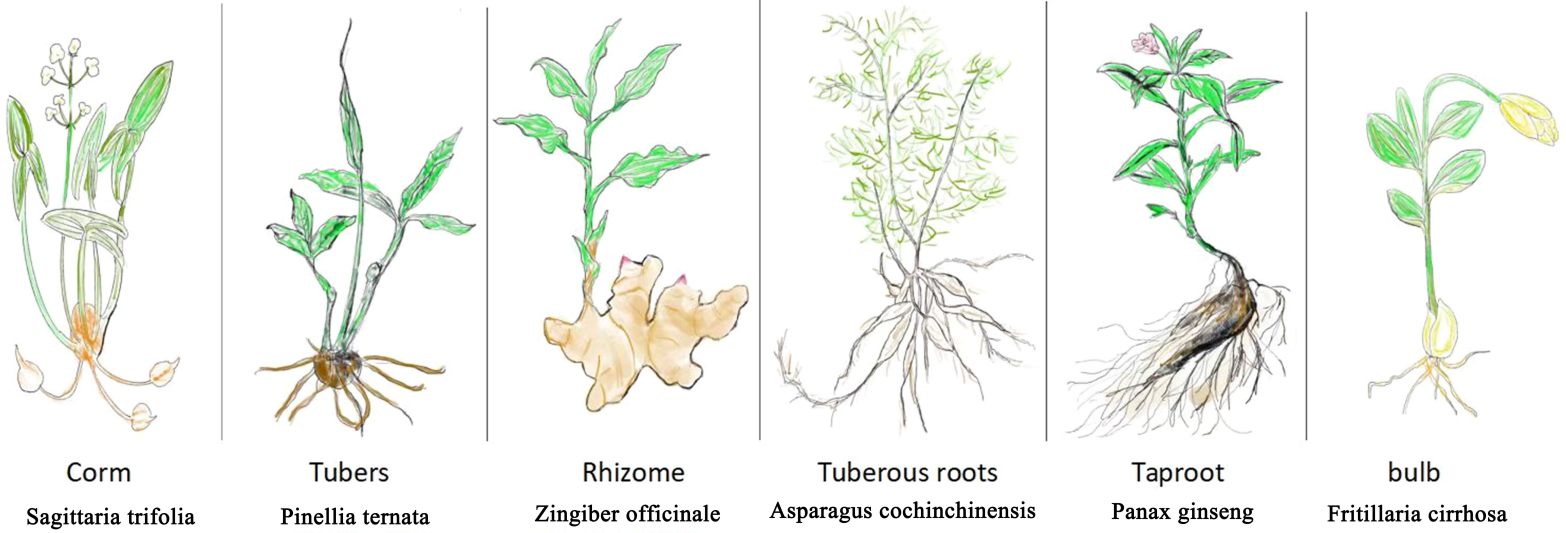
Examples of different swollen roots in medicinal plants. From left to right, the image shows corms, tubers, rhizomes, tuberous roots, taproots, and bulbs. Representative species include *Sagittaria trifolia* (corm), *Pinellia ternata* (tuber), *Zingiber officinale* (rhizome), *Asparagus cochinchinensis* (tuberous root), *Panax ginseng* (fleshy taproot), and *Fritillaria cirrhosa* (bulb). Green parts indicate aerial shoots, and brown parts represent underground organs.

Underground stems include corms, tubers, rhizomes, and bulbs. Corms are formed by the thickening of basal stem nodes, as observed in *Colocasia esculenta* (taro), *Heleocharis dulcis* (water chestnut), and *Sagittaria trifolia* (arrowhead) (Zhang et al., 2024). Representative examples of these diverse types of storage organs are shown in **Figure 1**. Tubers develop through stolon enlargement and occur in species such as *Corydalis yanhusuo* and *Sparganium stoloniferum* (Yang et al., 2024). Rhizomes are horizontally growing underground stems, with *Zingiber officinale* (ginger) representing a typical example (Xing et al., 2022). Bulbs are modified underground stems, as exemplified by *Fritillaria cirrhosa and Lilium lancifolium*. Bulb formation may occur through multiple developmental routes, including germination from the seed embryo, development from leaf axils, growth from adventitious buds at the stem or bulb base, and emergence from axillary buds of the parent bulb or adventitious shoots from bulb scales (Xu et al., 2020; Bellinazzo et al., 2025).

Swollen roots mainly include tuberous roots and fleshy taproots. Tuberous roots are storage organs derived primarily from adventitious or lateral roots and lack both hypocotyl and stem tissues (Abelenda et al., 2019). Representative species include *Pleuropterus multiflorus*, *Pseudostellaria heterophylla*, *Aconitum kusnezoffii*, and *Ampelopsis japonica*. In contrast, fleshy taproots develop through thickening of the primary root, usually involving the embryonic axis and a short upper stem internode, whereas lateral roots remain relatively thin and short. Representative species include *Panax ginseng*, *Angelica dahurica*, *Angelica sinensis*, *Heracleum sinensis*, *Peucedanum praeruptorum*, and *Saposhnikovia divaricate* (Kim et al., 2016; Zierer et al., 2021).

In *Crocus sativus*, corm development is typically divided into three stages. The initial stage is characterized by swelling at the base of the underground stem, driven primarily by active cell division and carbohydrate accumulation, particularly starch. During the subsequent expansion stage, rapid cell enlargement becomes dominant, leading to significant increases in corm size (Jose-Santhi et al., 2024). In *Lilium* spp., bulb enlargement is spatially and cell-type specific, occurring mainly in storage tissues rather than throughout the underground organ. This process is regulated by localized hormone and sugar signaling, which establishes growth centers and promotes anisotropic cell expansion through cytoskeletal organization, cell-wall remodeling, and osmotic regulation (Hao et al., 2023). In *Solanum tuberosum*, underground organ formation is modulated by photoperiodic cues, with members of the *PHOSPHATIDYLETHANOLAMINE-BINDING PROTEIN* (*PEBP)* family, such as *SELF-PRUNING6A (SP6A)*, inducing localized bulb or tuber initiation (Plunkert et al., 2024). From an evolutionary perspective, localized bulb enlargement may represent an optimized strategy that enhances carbohydrate storage efficiency while reducing energy costs and maintaining mechanical stability (Leverett et al., 2023). Root enlargement occurs preferentially in regions with persistent or reactivatable vascular cambium. In species such as *Panax ginseng* and *Ipomoea batatas*, primary roots and hypocotyl–root transition zones retain meristematic competence, whereas lateral roots often lack sustained cambial activity. Thus, anatomical predisposition determines the spatial specificity of storage-root formation (Nieminen et al., 2015; De Rybel et al., 2016).

In *Ipomoea batatas*, root development involves extensive histological changes that are largely coordinated by the vascular system through the combined activities of primary and secondary meristems (Noh et al., 2010). The primary meristem contributes to root primordium formation, whereas the secondary meristem promotes root thickening by increasing both cell number and cell size. Anatomical studies of developing roots have greatly advanced our understanding of the mechanisms underlying root enlargement.

In many medicinal roots, organ enlargement results from coordinated cell division in the cambial region and starch accumulation (Tanaka, 2016; Khanam et al., 2023). Tubers enlarge through extensive cell proliferation in the cortex, medullary ring, and pith (Hou et al., 2024). In *Raphanus sativus L.*, storage roots derived partly from the hypocotyl region undergo secondary thickening through cambial activity, with xylem differentiating inward and phloem outward; in many cases, xylem production predominates (Zaki et al., 2012). Several medicinal roots commonly cultivated in northeastern China, including *Achyranthes bidentata*, *Isatis tinctoria*, *Astragalus membranaceus*, and *Platycodon grandiflorum*, exhibit distinct anatomical patterns during root thickening. Seedlings of *Achyranthes bidentata* show a characteristic root developmental pattern in which the root neck, root body, and root tail originate from distinct anatomical regions along the axis. Root thickening in this species is mainly associated with the proliferation and differentiation of tertiary vascular and parenchymatous tissues, which contribute significantly to the overall enlargement of the root system and the accumulation of secondary metabolites such as triterpenoid saponins (Li and Hu, 2009). In *Isatis tinctoria* seedlings, the cotyledonary node exhibits an intermediate elongation type. The central vascular cylinder shows an intermediate initiation pattern and originates from two separate primordia. The root neck develops from the upper cotyledonary node, whereas the root body is derived from the lower node and hypocotyl. The primary root contributes only partially to taproot thickening (Speranza et al., 2020). In *Astragalus membranaceus* seedlings, the cotyledonary node corresponds to the internode elongation type, characterized by an unequal diarch stele. The root neck is derived from the cotyledonary node, and the taproot body originates from the hypocotyl transition zone. Unlike typical fleshy roots, thickening in *Astragalus* depends largely on highly lignified secondary xylem. In young seedlings, the cotyledonary node exhibits interbranch elongation with a triarch stele, where the short node forms the root neck and the hypocotyl forms the root body (Campbell et al., 2018; Ben-Targem et al., 2021).

In *Panax pseudoginseng*, primary root enlargement depends largely on secondary meristem activity and exhibits marked seasonal variation. In March, the vascular bundles remain poorly developed. From May to July, the vascular cambium becomes highly active, producing secondary phloem outward and secondary xylem inward, thereby driving rapid radial growth. By October, root thickening continues but at a reduced rate (Li et al., 2019). Similarly, in *Ficus religiosa*, tuber initiation is triggered by division and differentiation of the secondary meristem. The continuous cell division in the secondary parenchyma and ground parenchyma initiates thickening (Liu et al., 2023). In addition, anatomical studies of *Ficus ginseng* tubers have revealed clear differences between non-enlarged and enlarged roots. In non-enlarged roots, cells largely retain primary anatomical characteristics, whereas enlarged roots display secondary growth features. Activation of the vascular cambium and cork cambium induces cell division, leading to the formation of secondary vascular tissues and periderm layers, consequently, root thickening. In enlarged tubers, increased cell division in the secondary xylem and parenchymatous tissues further contributes to tuber expansion. In addition, parenchyma cells accumulate abundant starch grains, supporting the storage function of enlarged roots (Liu et al., 2023).

Such structural differences are important not only from taxonomic and morphological perspectives but also as a scientific basis for the traditional identification and quality evaluation of root- and rhizome-derived medicinal materials in ethnopharmacology. In traditional identification practices, the quality of roots and rhizomes is often assessed by specific internal structures that reflect microscopic changes associated with cell differentiation and secondary metabolite accumulation influenced by genetic and environmental factors. For example, in *Astragalus membranaceus* (Huangqi), the “chrysanthemum heart pattern,” a key feature used in traditional identification, corresponds to the radial texture formed during secondary growth of the primary root. This pattern results from the alternate arrangement of xylem and phloem rays, fibers, and parenchyma cells, indicating strong transport and storage capacity (Peng et al., 2017). By contrast, according to the *Pharmacopoeia of the People’s Republic of China* 2020 Edition, the “lion’s head pattern” observed in *Codonopsis* species is associated with prominent stem scars and bud formation at the rhizome junction, which may indicate advanced plant age and relatively low nutritional status. The “cinnabar spots” observed in some medicinal materials, such as *Atractylodes* species, are associated with secretory structures and the accumulation of volatile compounds, which contribute to their traditional quality evaluation. Structural abnormalities can also serve as important diagnostic features in traditional identification. For example, the “cloud-brocade pattern” in the cortex of *Polygonum multiflorum* rhizomes and the “compass pattern” in *Phytolacca* roots are both associated with abnormal vascular cambium activity during development (CPC, 2020). The former is a characteristic feature used for species identification, whereas the latter is important for recognizing toxic medicinal materials and assessing their quality. In *Fritillaria cirrhosa*, the commercially important “moon-embracing pattern” results from two markedly unequal fleshy scales enclosing the bulb. Together, these traditional diagnostic features have clear anatomical and developmental relevance and provide a scientific basis for the quality evaluation of medicinal underground organs (CPC, 2020).

## 3 External factors influencing root expansion

Root development and subsequent enlargement are complex processes governed by both genetic and environmental factors. External cues that directly influence root development, including the thickening stage, include temperature, light, water availability, soil aeration, nutrient and mineral supply, and heavy metal stress.

### 3.1 Physical factors

In *Solanum tuberosum*, favorable temperatures enhance vascular cambium activity and promote tuberous root formation, whereas excessively high temperatures inhibit root initiation and low temperatures generally suppress root growth. A large diurnal temperature range may further stimulate root development (Zinta et al., 2024). The *CONSTANS-LIKE1* (*StCOL1*) gene regulates tuberization under photoperiodic conditions in *Solanum tuberosum*. In contrast, the expression of tuberization inhibitors, including the *SHORT VEGETATIVE PHASE5 gene (StSP5G)* and *StCOL1*, is induced, thereby impairing tuber formation (Park et al., 2022). Heat stress can also inhibit root cell division by disrupting microtubule function and the homeostasis of reactive oxygen species (ROS). In contrast, low temperature alters root architecture by modulating hormone levels, particularly through auxin- and cytokinin-related pathways (Kazan, 2015; Li and Howell, 2021). Cytokinin treatment has been reported to increase the rate of root cell division in *Arabidopsis thaliana*, resulting in a 20–30% increase in root cell number; however, excessive cytokinin concentrations inhibit further storage-root enlargement (Tiwari et al., 2023). Low-temperature stress also induces the expression of stress-responsive genes, such as *DEHYDRATION-RESPONSIVE ELEMENT-BINDING PROTEIN2.1* (*DREB2.1)*, as well as root hair-related genes, including *ROOT HAIR DEFECTIVE3 (RTH3)* and *ROOT HAIR DEFECTIVE6 (RTH6)*, which regulate root hair elongation under cold conditions, as reported in model and crop species such as *Arabidopsis thaliana* (Berdion et al., 2025).

In *Arabidopsis thaliana*, adequate oxygen availability is essential for root growth, whereas oxygen deficiency can lead to excessive accumulation of ROS and oxidative damage in root cells. Reoxygenation treatment may help maintain ROS homeostasis and reduce hypoxia-induced oxidative injury in roots (Liu et al., 2022). In addition, oxygen availability may promote root cell expansion by regulating cell-wall acidification and membrane transport activity. Because cell-wall acidification is critical for radial expansion, oxygen enrichment may facilitate this process through improved regulation of the extracellular redox cycle (Mellidou and Kanellis, 2024). Together, these mechanisms contribute to root thickening and underground organ enlargement.

Elevated carbon dioxide (eCO_2_) has a significant impact on root development. In *Eucalyptus* spp., at eCO_2_, plants increase the amount of photosynthesis-derived assimilates allocated to their roots. Root carbon increment in agricultural systems under eCO_2_, for instance, was found to be considerably high (≈17.2 t C ha), with over 60% of the carbon allocated to underground organs (de Oliveira et al., 2025). Additionally, eCO_2_ affects root anatomical structure in terms of cell wall thickness (Khandal et al., 2025). eCO_2_ also induces gene expression for plasma membrane intrinsic protein 1;3 (*PIP;3*), which increases water transport-related activities that indirectly result in root development (Pérez-Martín et al., 2024). Light and photoperiod are also important factors. Longer days increase the efficiency of photosynthesis and contribute more to root development (Spaninks and Offringa, 2023). The leaves perceive the difference in day length and transmit the information to the shoot tip via the phloem. In short days, tubers are initiated at the tip of the stolons. Major genes *PHYTOCHROME B (PHYB), FLOWERING LOCUS T* (*FT)*, *CONSTANS (CO)*, and *GIGANTEA* control tuber formation, with *CO-FT* being critical (Inui et al., 2010). Excessive light can impair root growth. During the summer, high light saturates stomata, represses photorespiration, and reduces assimilate formation, hence repressing root growth (Miotto et al., 2021). In *Manihot esculenta*, soil water availability is a critical factor in root extension. Well-irrigated soil enables greater root diameters and a higher crown-to-root ratio. When water is inadequate, it reduces root extension but increases root abnormalities. Excessive water limits oxygen diffusion to the root and represses root extension. During drought, plants allocate more dry matter to extension rather than to thickness. Under drought conditions, the dry weight of storage roots decreased by 30–50%, while under moderate irrigation, root enlargement rates increased by 1.4–1.7 times (Joseph et al., 2016). In *Panax ginseng*, forest-grown plants showed up-regulation of starch and sucrose metabolism genes and down-regulation of lignin biosynthesis genes compared with field-grown plants, which was subsequently associated with increased accumulation of sucrose, fructose, and ginsenosides, as well as greater root diameter and root weight (Eo and Park, 2019).

Most medicinal roots undergo pronounced enlargement during spring and autumn, whereas root growth is relatively limited in other seasons. Enlargement of underground storage organs is often regulated by photoperiod (Jackson, 2009). In some tuber-forming species, such as *Solanum tuberosum*, short-day conditions are required for tuber initiation, and seasonal changes in day length during spring and autumn may activate related regulatory genes, including members of the PEBP family. Specific PEBP members, such as *FT* homologues, are up-regulated under short-day conditions, and their overexpression can promote tuber formation even under non-inductive long-day conditions (Plunkert et al., 2024). Underground storage organ enlargement is also an important adaptive strategy that enables plants to survive adverse environmental conditions, such as drought and low temperature. Seasonal changes in temperature and soil moisture during spring and autumn may induce the expression of genes related to auxin response, sugar transport, and meristem activity, including *ARFs*, sugar transporter genes, and *WUSCHEL-RELATED HOMEOBOX* (*WOX)* genes, thereby promoting cell proliferation and expansion (Klimešová and Herben, 2024). *ARFs* and cytokinin oxidase genes may promote enlargement by regulating cell division and expansion. Sugar transporter genes directly influence carbohydrate accumulation in storage organs. In addition, *WOX* genes and *CLAVATA3/ESR-related (CLE)* peptides regulate cell proliferation and differentiation within the vascular meristem, thereby facilitating storage organ enlargement (Plunkert et al., 2024; Wu et al., 2025). Plants also adjust carbon allocation in response to phenological changes. An earlier start to the spring growing season can enhance root carbon allocation in warm climates, whereas an extended autumn growing season may increase root carbon accumulation in arid climates (Ren et al., 2024). Overall, the response of medicinal root growth to environmental conditions involves complex, multidimensional regulatory networks. Key external environmental factors influencing root enlargement in medicinal plants, including temperature, light, and soil nutrients, are summarized in **Table 1**.

**Table 1:** Key external environmental factors influencing root expansion in medicinal plants.

External environmental factors	Mechanism
Temperature	Air temperature and root zone temperature jointly affect plant growth parameters and photosynthetic characteristics. The high temperature promotes root elongation through the brassinolide signaling pathway (particularly down-regulation of receptor BRI1), and this process is independent of the auxin pathway. Low temperatures (1-5 °C) significantly inhibit apical cell elongation and lignification, reducing the formation of secondary roots. Light intensity regulates the response of *Arabidopsis thaliana* roots to high temperatures through the TAA1 gene, while the synergistic effect of phosphorus deficiency and high temperatures in the root zone reduces root viability. The root system can sense temperature changes on its own and regulate growth through cell division and elongation, with auxin, cytokinin and brassinolide playing a central role in the temperature response.
Oxygen	Adequate oxygen supply significantly promotes root morphology development, including increasing root surface area (149.7%), root length (181.2%), bifurcations (198.4%), and root tips (165.4%). Secondly, in hypoxic conditions, plants enhance their oxygen transport capacity by forming aerenchyma and increasing the number of adventitious roots. For example, reed (P. australis) showed shorter stems, more tillers, and more adventitious root formation under hypoxic treatment. Plant hemoglobin 1 (Pgb1) regulates hypoxic adaptation by removing nitric oxide (NO) and affects auxin transport (PAT), thereby altering the bending direction of the root system under hypoxic conditions. Oxygen also regulates root development by influencing reactive oxygen species (ROS) levels. For example, bacterial inoculation in corn roots stimulates auxin synthesis by raising ROS levels, which in turn promotes an increase in lateral root count and root dry weight. Cell wall thickening structures, such as thickening of the outer cortex, prevent oxygen loss and enhance root penetration, helping plants adapt to stress environments.
CO2	High concentrations of CO2 (e[CO2]) promote overall plant growth by enhancing photosynthesis, including increasing underground carbon distribution and nitrogen absorption, thereby indirectly promoting root swelling. The root system can absorb inorganic carbon (such as bicarbonate) through anion channels, transport it via xylem to the aboveground, convert it to CO2 by carbonic anhydrase and fix it by RuBisCo, a process that not only promotes photosynthesis but also provides an additional carbon source for root growth. Under low-nitrogen conditions, e[CO2] can drive the development of the endodermis of the root system, adapting to nutrient acquisition requirements through morphological and anatomical changes such as an increase in lateral roots and an increase in root diameter. CO2 may also affect root water transport and cell expansion by modulating aquaporin genes (such as PIP1;3) and membrane polarization signals (such as SLAH1/3, AHA2). The direct effect of CO2 on root biomass may be weaker, and its effect often interacts with environmental factors such as water status or temperature, for example, CO2 can indirectly maintain root growth by reducing transpiration to alleviate water stress under drought conditions.
Light exposure time	Light duration (photoperiod) affects plant root swelling through multiple mechanisms. Photoperiodic changes regulate root growth rates, and the pattern of changes in root elongation rates remains consistent with a fixed photoperiod even under different temperature conditions, indicating that light is not a direct trigger for root elongation. The light signals are mainly perceived by photoreceptors in the above-ground parts (such as photosensitive pigment PHY and cryptochrome CRY) and transmitted to the root system through mobile signal molecules such as hormones (such as auxin) to regulate the development of the main and lateral roots. Photoperiodic changes affect the synthesis, transport and distribution of endogenous plant hormones such as auxin, which in turn affect the expansion and division of root cells. Light also induces the accumulation of flavonoids in the root system, which may inhibit root growth by regulating reactive oxygen species levels or cytoskeletal tissue. The response of roots to photoperiod is species-specific. For example, photoperiod sensitivity in short-day plants (such as buckwheat) significantly affects their root development.
Light intensity	Light intensity regulates root swelling through a complex network of interactions between photoreceptors and auxin signaling pathways. Different light qualities (such as blue and red) affect root development through specific photoreceptors (such as CRY1 and phyB): blue light promotes root growth through cryptochrome signaling, while red light receptor phyB inhibits auxin signaling by stabilizing AUX/IAA proteins. The light signal travels from the aboveground part to the root system, regulates root growth by activating the HY5 transcription factor, while light induces the accumulation of flavonols in the root system to inhibit growth. Auxin, as a core regulator, has its synthesis, transport and signal transduction all affected by light intensity. Low light intensity significantly inhibits lateral root development, while light-induced changes in auxin distribution regulate root configuration by modulating transcription factor cascade reactions such as ARF-LBD. Light signals work in synergy with sugar signals to integrate environmental signals and endogenous hormone balance through pathways such as TOR-E2FA.
Soil moisture content	Under conditions of abundant moisture, roots tend to extend horizontally (as in shallow wide soil) or vertically (as in deep narrow soil) to make full use of water resources. When soil moisture content is reduced, certain plants (such as the corn variety FR697) can extend root elongation by maintaining the rate of cell generation and regulating the rate of tissue expansion, while the diameter of primary lateral roots increases (by up to 2.3 times volume) as water stress intensifies. In addition, anatomical features such as the area ratio of the root cortex to the top column change adaptively with the gradient of soil moisture content. Under extreme drought conditions, the overall expansion capacity of the root system is limited, but some plants balance water absorption by enhancing root development. Water stress also affects root meristematic activity by modulating cell wall yield properties, osmosis regulation, and signaling molecules such as abscisic acid. Root water absorption changes soil water-holding capacity by creating negative pressure and inducing microfissures in the rhizosphere soil, which in turn feedback regulates the root swelling process.
Land minerals	Soil minerals affect plant root swelling through multiple mechanisms: iron (Fe) and aluminum (Al) binding to malic acid secreted by roots at concentrations of 10μM cause cell wall (CW) hardening and root extension arrest, while a single metal ion only leads to an increase in hardness but not enough to inhibit growth. Silicon (Si) can reduce the inhibition of root elongation caused by aluminium toxicity after being absorbed through a transporter, but the specific protective mechanism remains unclear. The addition of calcium (Ca) and magnesium (Mg) changes the aluminium activity threshold, significantly affecting root elongation in leguminous and gramineous plants. In addition, soil compaction reduces the accumulation of mineral elements such as phosphorus and potassium by inhibiting isocitrate dehydrogenase and cytochrome c oxidase activity, resulting in reduced root cell size and blurred boundaries. Low iron conditions accelerate root growth in *Arabidopsis thaliana*, while low phosphorus or low zinc inhibits growth, suggesting that different mineral deficiencies trigger differentiated root growth adaptation strategies. Aluminum poisoning can also be mitigated by boron (B) intervention, which involves reducing the lignification of cell walls and enhancing resilience.
Compost	Composting improves rhizosphere nutrient availability, increases soil organic carbon content, pH and electrical conductivity, and enhances enzyme activity and soil respiration; Enhance microbial activity, stimulate the growth of rhizosphere microorganisms and enzyme activity, thereby increasing rhizosphere auxin concentration and promoting root development. By inducing biological processes related to organ morphogenesis and development, such as root crown development, lateral root formation, and post-embryonic root morphogenesis; Altering root morphological characteristics, increasing root length, volume, surface area and the number of root tips, while possibly reducing root diameter; Reduce lipid peroxidation levels in the root membrane and improve plant stress resistance; Alter root water use efficiency through substances such as humic acid and promote lateral root elongation; Regulating changes in rhizosphere pH affects microbial utilization of root secretions and mineralization of soil organic carbon.
Nitrogen fertilizer	The level of nitrogen fertilizer application significantly affects root biomass, length, and diameter, where an appropriate amount of nitrogen fertilizer (such as 150 kg/ha) promotes root growth, but excessive application inhibits root development instead. In terms of nitrogen form, nitrate (NO3-) stimulates primary root growth by promoting cytokinin signaling and meristematic activity, while ammonium (NH4+) inhibits cell expansion and division, resulting in delayed root elongation. Short-term low nitrogen conditions trigger root elongation to enhance nutrient absorption, a process that involves key regulation of nitrate signaling components. Combined application of nitrogen fertilizer with organic fertilizer (such as sheep manure) promotes root development by improving soil physicochemical properties and enzyme activity, while simple nitrogen reduction inhibits root vitality. At the molecular level, the nitrogen signaling pathway affects root configuration by regulating the balance of auxin and brassinolide (BR), where NO3- relies on the BR signaling pathway to promote root elongation, while NH4+ restricts root growth by inhibiting BR and auxin signaling.
Microbial communities	Microbial communities are capable of regulating lateral root development through pathways that operate independently of classical plant hormones like auxin. For instance, secondary metabolites derived from microorganisms can directly or indirectly modulate the synthesis and signaling of plant hormones—such as auxin and cytokinin—thereby influencing root expansion. Additionally, interactive networks within the rhizosphere, including associations among bacteria, fungi, and protists, may alter root morphology via nutrient competition or synergistic relationships. Furthermore, microbial activity enhances nutrient availability—including nitrogen, phosphorus, and potassium—in the rhizosphere through processes such as organic matter decomposition, biological nitrogen fixation, and phosphorus solubilization. These contributions indirectly support the accumulation of root biomass.

### 3.2 Chemical factors

In *Arabidopsis thaliana*, exogenous glutamate (Glu) can stimulate radial expansion, increase seedling height, and increase leaf color intensity (Ma et al., 2020). Salt stress exerts distinct effects on root architecture, suppressing primary root elongation but increasing lateral root density (van Zelm et al., 2023). Genetically, natural variation is associated with differences in radial expansion responses in salt-treated plants (Zhang X et al., 2023). Mineral nutrients play important roles in influencing root morphology. Iron (Fe) deficiency induces uptake responses in monocots and dicots (Joon et al., 2025). Fe is also responsible for root architecture when nitrogen (N), phosphorus (P), or potassium (K) is deficient (Ahmad et al., 2025). Root development with respect to the primary root is influenced by the availability of calcium (Ca) (Silva et al., 2024). Phosphorus deficiency promotes the development of lateral roots and root hairs (Lu et al., 2024). Phosphorus addition increases root tip number, diameter, and density, while microbial relationships range in their effects (Cheng et al., 2025; Choi et al., 2025). Potassium addition increases root length and branching; for example, in *Zea mays*, the absence of phosphates led to a 25.4% improvement in K use efficiency (Cheng et al., 2025). Environmental factors also affect metabolite production. Heavy metals restrict root expansion. Cadmium (Cd) inhibits root growth, although root exudations can chelate metals and reduce their bioavailability (Lin et al., 2025). Lead (Pb) and zinc (Zn) stress can be alleviated by indole-3-butyric acid (IBA), which increases root surface area and lateral root number (Chen et al., 2025).

### 3.3 Biological factors

**Figure 2:**
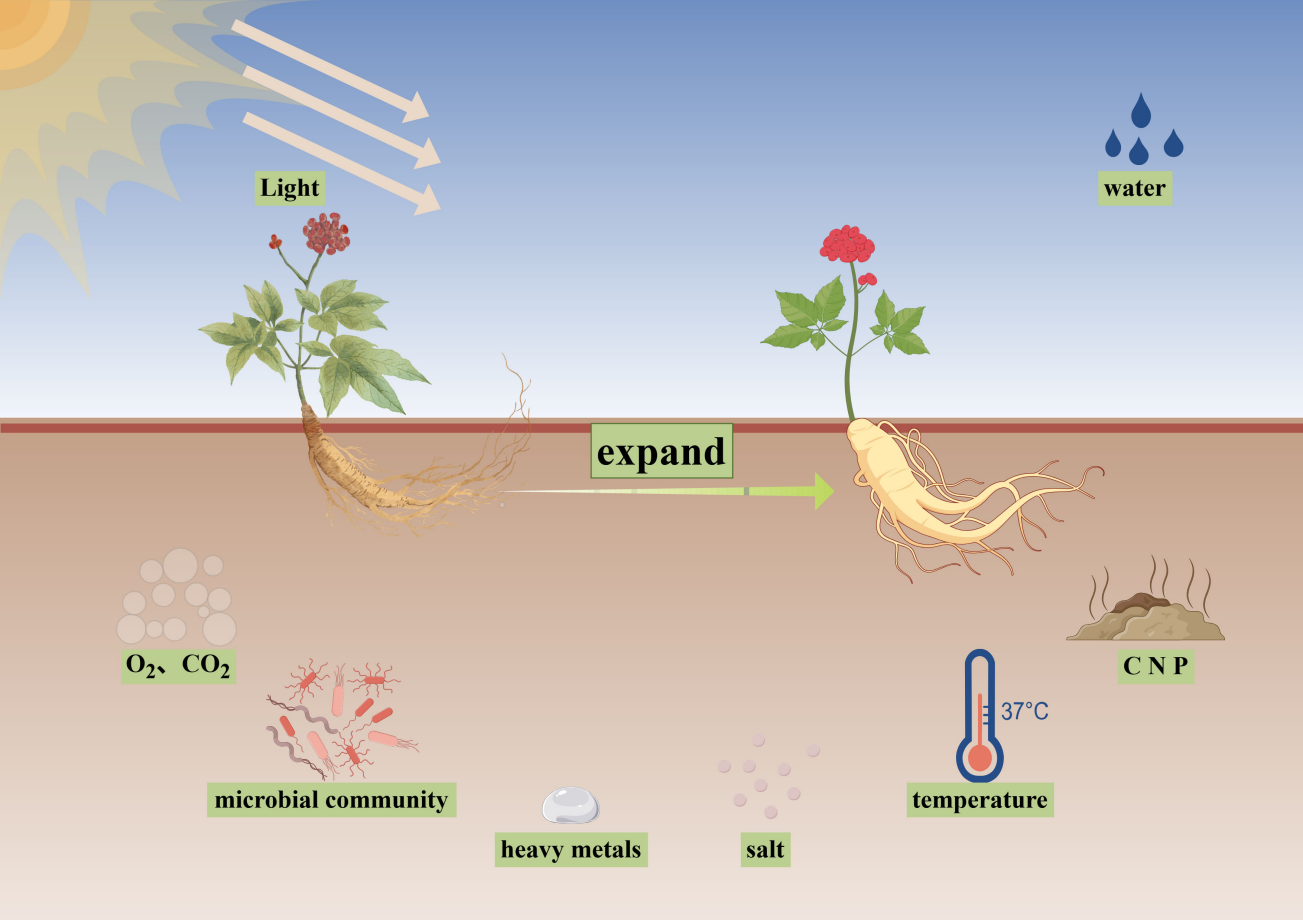
Environmental factors regulating root expansion in medicinal plants. This image summarizes the main environmental factors influencing root thickening. Above-ground factors (e.g., light intensity and water supply) regulate photosynthesis and the production of assimilates, which are transported to underground organs. Subsurface factors (including O_2_, CO_2_, nutrient supply (C, N, P), temperature, soil microorganisms, heavy metals and salinity) influence root metabolism, cell proliferation and storage compound accumulation, thereby affecting root thickening. Arrows indicate nutrient transport and root expansion, and yellow areas represent storage tissues rich in carbohydrates or secondary metabolites. Icons above the soil surface denote above-ground factors, while symbols within the soil indicate underground abiotic and biotic influences. C, carbon; N, nitrogen; P, phosphorus; O_2_, oxygen; CO_2_, carbon dioxide.

Soil microbes also play an indirect but vital role in root expansion. Root-associated microbes in *Solanum lycopersicum* can enhance iron uptake under Fe-deficient conditions by mobilizing soil Fe (Zhu et al., 2025). Beneficial microorganisms, such as *Streptomyces* and *Mortierella*, can suppress *Fusarium oxysporum*, thereby reducing root rot and promoting root enlargement (Liu Y et al., 2025). In addition, microbial secondary metabolites may induce systemic resistance and stimulate root growth (Mukherjee et al., 2024). Root exudates, including oxalic acid, can alleviate salt stress by enhancing the expression of photosynthesis-related genes and improving antioxidant capacity (Chen et al., 2024). Thus, root expansion ultimately reflects the integration of environmental signals at the molecular level, as detailed in Section 3. The major environmental factors and their proposed modes of action are summarized in **Figure 2**.

## 4 Molecular mechanisms of plant root expansion

Root elongation and thickening involve complex biological processes that integrate growth, morphogenesis, environmental adaptation, and photoassimilate accumulation (Kondhare et al., 2021). Enzymatic activity and transcriptional reprogramming play essential roles in root and rhizome development by regulating endogenous hormone signaling, lignification, starch biosynthesis, and metabolic processes (Gui et al., 2011). During radial expansion, active formative cell division is typically associated with reduced lignification. At the expansion stage, the cessation of longitudinal cell growth, the shift from longitudinal elongation to radial expansion, and sustained cell proliferation collectively promote thickened root formation. Thus, vascular cambium activity regulates the initiation of root expansion, whereas the balance between cambial activity and lignification determines the efficiency of thickened root development (Chiatante et al., 2018).

To better understand the molecular mechanisms underlying root swelling, it is essential to first clarify the spatial relationship between the root swelling zone and metabolite storage sites at the tissue level. In most storage roots, radial expansion originates primarily from the vascular cambium and its derivatives, particularly secondary xylem and adjacent parenchyma. Active cell division and cell expansion in these tissues constitute the major structural basis of the enlarging organ. However, the primary sites of metabolite accumulation may either overlap with these growth zones or occur in nearby specialized tissues, depending on the metabolite type and plant species (Wang et al., 2017). For example, starch, soluble sugars, and certain water-soluble polysaccharides are commonly stored in enlarged xylem parenchyma cells or other storage parenchyma cells, which also directly contribute to root thickening (Carluccio et al., 2022). In contrast, lipophilic or highly specialized secondary metabolites, such as volatile oils, oleoresins, and secreted phenylpropanoid-derived compounds, are often concentrated in discrete secretory structures, including oil ducts, secretory canals, oil cells, and glandular cavities, rather than in the main proliferative tissues themselves (Wang H et al., 2024; Fu et al., 2026; Xiang et al., 2026). For example, the volatile oils of *Angelica sinensis* are primarily stored in the oil ducts (Li et al., 2023). In *Arabidopsis thaliana*, stress-related compounds derived from *Astragalus* polysaccharides have been reported to accumulate in tissues surrounding the xylem vessels, including ray parenchyma cells (Loupit et al., 2023). Therefore, root thickening should be viewed as a process that establishes both growth and storage domains. In some species, these domains largely overlap, whereas in others they are anatomically distinct but developmentally coordinated. This spatial coordination may explain why cambial activity and parenchyma proliferation are closely associated with metabolite accumulation. It also suggests that the molecular regulation of root thickening involves tissue-specific control of both growth and storage functions, likely mediated by coordinated hormonal signaling and gene expression networks (Oltmanns et al., 2006; Dong et al., 2019).

### 4.1 Changes in endogenous plant hormones and related genes that affect root expansion

Plant growth, organogenesis, morphogenesis, and expression of characters are governed by plant hormones. The principal plant hormones are auxins (indole-3-acetic acid, IAA), gibberellins (GA), cytokinins (CTK), abscisic acid (ABA), ethylene (ETH), jasmonic acid (JA), and salicylic acid (SA). Different hormones contribute differently to root growth; however, the total number of roots produced is linked to their internal levels.

Auxin IAA plays a central role in regulating tuber and caudex biosynthesis in species such as *Panax ginseng*. IAA regulates tuber initiation and expansion as a function of the *AUXIN RESPONSE FACTOR (ARF), AUX/IAA, GRETCHEN HAGEN3 (GH3)*, and *SMALL AUXIN UP RNA (SAUR)* gene families that include *IAA13, IAA16*, and *ARF7*. IAA treatment significantly promoted root enlargement, with root diameter increasing by approximately 18–35% (Lomin et al., 2023; Liangdeng et al., 2024). ARFs are plant-specific transcription factors that directly bind auxin response elements (AuxREs) to activate or repress downstream gene expression. In *Arabidopsis thaliana*, ARF activity is dynamically regulated by AUX/IAA proteins: in the absence of auxin, AUX/IAAs (e.g., *IAA9*) interact with ARFs (e.g., *ARF7*) to inhibit their function; upon auxin perception, AUX/IAAs are degraded, releasing ARFs to activate target genes (such as *PIN-FORMED1 (PIN1)*) and promote cell division and expansion (Hu et al., 2022). AUX/IAA proteins (such as *IAA13* and *IAA16*) act as repressors of auxin signaling; mutation of *IAA13* results in reduced numbers of lateral roots (LRs) and lateral root primordia (LRPs), along with delayed radial expansion, indicating that it regulates organ enlargement by modulating the cell division cycle (Yamauchi et al., 2024). GH3 enzymes (e.g., *GH3.5* and *GH3.17*) conjugate IAA to amino acids, thereby reducing free auxin levels and fine-tuning signaling strength (González et al., 2024). In *Pueraria lobata*, at a biochemical level of control, IAA regulates the tryptophan metabolism pathway (ko00380), which targets genes *YUCCA FLAVIN MONOOXYGENASE10* (*YUCCA10)* and *COBRA* and induces expansion genes *CELL DIVISION CYCLE48 (CDC48)* and *EXPANSIN (EXP)* (Liangdeng et al., 2024).

GA modulates root and tuber growth in species such as *Solanum tuberosum* and *Oryza sativa* by regulating active GA levels via GA20 oxidase (GA20ox), thereby affecting primary root growth and lateral root development (Zhao L et al., 2024). In GA signaling, DELLA proteins bind to transcription factors such as SCL3 to regulate cell division in root meristematic tissues (Xing et al., 2025). However, GA also interacts with other hormones; for instance, it inhibits crown root growth in *Oryza sativa* by binding to the OsGER4 protein, which negatively interacts with JA (Meng et al., 2024). Additionally, degradation of GA by P450 enzymes such as *CYP714A1* can inhibit tuber growth (Jiang et al., 2023). GA also acts as a concentration-mediated regulator, promoting growth at lower concentrations but inhibiting it at higher concentrations at the root level (Meng et al., 2024).

Trans-Zeatin Riboside (t-ZR) is the nucleosylated form of cytokinins (CKs) that governs long-distance transport; t-ZR is synthesized in the roots and transported to the aboveground parts, whilst its distribution within the roots is regulated by transport proteins. In *Arabidopsis thaliana*, *AtENT3* is involved in the long and short-distance transport of t-ZR, directly influencing the overall CK concentration in the roots (Nedvěd et al., 2025). During tuber development, t-ZR accumulates significantly in specific regions, such as the swelling tuber and the root tip. In *Solanum lycopersicum* research, exogenous treatment that elevates t-ZR levels has been shown to facilitate rapid shoot and root growth to adapt to new growing conditions (Díaz-López et al., 2024).

Phytohormones such as ABA, ETH, methyl JA, and SA are vital regulators of radial expansion and primarily act through hormonal-gene interactions. In *Arabidopsis thaliana*, ethylene acts as a sensor of soil friction stress and promotes root tip expansion by activating ABA and auxin signaling pathways (Huang et al., 2022). Similarly, JA regulates roots by inhibiting meristematic activity under salt stress while activating genes involved in crown root development (Muzaffar et al., 2024). In *Arabidopsis thaliana*, ABA, SA, and JA signaling can converge through *MYC2*-mediated regulatory modules. MYC2 functions as a central bHLH transcription factor in JA signaling and participates in crosstalk with ABA and SA-related pathways, thereby coordinating stress perception and adaptive root responses (Datta et al., 2024; Wan et al., 2025). Antagonism between JA and AUX contributes to root stress responses to salt stress. All hormonal regulators interact with downstream actors such as reactive oxygen species (ROS) and glutathione (GSH), which then adequately coordinate stress perception with active root development (Liu W et al., 2025).

In the tuberous roots of *Ficus ginseng*, the transcript levels of the cytokinin biosynthesis gene *ISOPENTENYLTRANSFERASE (IPT)* showed a progressive increase during the expansion of the roots, while the levels of the cytokinin degradation gene *CYTOKININ OXIDASE/DEHYDROGENASE* (*CKX*) showed a net decrease. Notably, *CKX* exhibited a biphasic expression pattern, with the gene down-regulated during the initial stages of expansion, followed by a period of up-regulation (Geng et al., 2023; Yamoune et al., 2024). Most hormone-linked genes peak in expression during the elongation and pre-expansion phases. This indicated that the respective stages are key initial points in tuber initiation and in the hormonal regulation of root development (Yamoune et al., 2024).

### 4.2 Genes involved in lignin formation influence plant root expansion

Lignin, a major secondary metabolite, forms a complex matrix in secondary cell walls and provides essential mechanical support to plant tissues. This structure is critical for maintaining tissue strength and water resistance (Xiao et al., 2025). Lignification is controlled by multiple genes involved in lignin biosynthesis and deposition, some of which also regulate root growth and thickening in medicinal plants.

Lignin biosynthesis mainly involves the phenylpropanoid pathway, cytochrome P450-mediated hydroxylation reactions, and downstream monolignol-specific modification steps. Phenylalanine serves as the primary precursor, which is converted through a series of enzymatic reactions into hydroxycinnamic acids, CoA esters, and finally monolignols. These monolignols are then transported to the cell wall, where they are polymerized to form lignin (Dixon and Barros, 2019). In *Arabidopsis thaliana*, CYP450-mediated metabolism is of primary importance for lignin metabolism. This metabolism is governed by several genes and by physiological responses to oxidative stress, leading to the accumulation of multiple metabolites. CYP450 members (e.g., *CYP84A1* and *CYP98A*) participate directly in lignin monomer hydroxylation (Zhao X et al., 2024). CYP450 enzymes are central to the phenylpropanoid pathway, supplying lignin precursors (El et al., 2021). CYP98A functions as a rate-limiting enzyme by catalyzing the 3-hydroxylation of coumaric acid esters to form intermediates for G- and S-lignin biosynthesis, while *CYP84A1 (F5H)* mediates the 5-hydroxylation of ferulic acid, promoting S-lignin formation (Zhao X. et al., 2024; Fang et al., 2025). Lignin precursors are further generated by shikimate or arogenate metabolism, phenylpropanoid metabolism, and lignin-specific modification reactions. Shikimate metabolism refers to the metabolism of photosynthetic commodities to yield the amino acids phenylalanine, tyrosine, and tryptophan. Phenylpropanoid metabolism further alters the three amino acids to hydroxycinnamic acids and hydroxycinnamic CoA esters. Lignin-modifying metabolism further converts hydroxycinnamic acid into a lignin precursor. Lignin precursors are then deposited into cell walls after dehydrogenation reactions, resulting in the formation of ethers, esters, and carbon-carbon bonds (Ma, 2024). Key enzymes and genes involved in these pathways include phenylalanine ammonia-lyase (PAL), cinnamate-4-hydroxylase (C4H), 4-coumarate:CoA Ligase (4CL), shikimate/quinate hydroxycinnamoyl transferase (HCT), coumarate-3-hydroxylase (C3H), caffeoyl-CoA 3-O-methyltransferase (CCoAOMT), cinnamoyl-CoA reductase (CCR), cinnamyl alcohol dehydrogenase (CAD), ferulate 5-hydroxylase (F5H), and caffeic acid O-methyltransferase (COMT). Lignin biosynthesis genes, such as *PAL*, *CCoAOMT*, and laccase (*LAC*), that are induced can strengthen root cell walls, thereby aiding the root’s supportive function during expansion. Interactions between lignin biosynthesis genes and hormonal genes can enhance roots’ ability to thrive under water-deficit stress (Wang Y et al., 2025).

Some enzymes involved in lignin function as key regulators of plant radial expansion, particularly in the phenylpropanoid biosynthesis pathway. Such enzymes include phenylalanine ammonia lyase (PAL), COMT, and CCR. Modulation of the expression levels or activities of genes involved in changes in lignin deposition may also influence plant radial expansion. An example of a key enzyme in the phenylpropanoid biosynthetic pathway of polyphenols and flavonoids is 4CL (Korany et al., 2026). In *Oryza sativa*, downregulation of 4CL activity, specifically *Os4CL3*, inhibits plant cell wall lignin deposition and plant root growth (Afifi et al., 2022). Evidence for the involvement of lignin biosynthesis in root development can also be found from transgenic experiments. Transcript analysis of the transgenics reveals strong induction of *PAL* and genes involved in lignin metabolism, such as *4-COUMARATE: COA LIGASE* (*4CL)*, *CINNAMATE-4-HYDROXYLASE* (*C4H*), and *CINNAMYL ALCOHOL DEHYDROGENASE* (*CAD*). All these genes are certainly involved in plant defense reactions. On the other hand, the transgenics exhibit increased activity of the starch-degrading enzyme β-amylase, thereby degrading starch throughout the plant (Gui et al., 2011). Taken together, these results indicate that genes involved in lignin biosynthesis act in concert to regulate root structure and function. This integration of metabolism, gene expression, and carbohydrate allocation emphasizes the importance of lignin for root growth. This study can serve as a basis for further research on the development of medicinal plant roots.

Lignin genes are intricately linked to the development of medicinal plant roots; however, preliminary results are still being generated. In *Panax ginseng*, several primary lignin-biosynthesis genes, including *PgCAD*, *PgCCR*, and *PgCOMT*, are suppressed during primary root development. However, overexpression of *PAL* genes is intricately associated with the accumulation of active molecules (Zhang M et al., 2023). Variations between the biomass of the root tubers and the amount of lignin further indicate that improper deposition of lignin may be responsible for tuber development due to continuous monocultures. In the context of *Rehmannia glutinosa*, the primary enzymatic genes responsible for lignin biosynthesis interact combinatorially under continuous monoculture. Specifically, modulation of the *CINNAMOYL-COA REDUCTASE6* (*RgCCR6)* genes has been shown to directly affect lignin biosynthesis and, therefore, impact the development of root tubers (Gu et al., 2021). In addition to its structural role, lignin biosynthesis can indirectly influence the distribution and accumulation of secondary metabolites by modifying cell-wall properties and tissue differentiation. In *Rehmannia glutinosa*, excessive lignification may restrict storage capacity and metabolic flux, thereby negatively affecting the accumulation of certain bioactive compounds in storage tissues (Wang et al., 2023).

### 4.3 Genes related to sucrose and starch metabolism affect plant root expansion

Root expansion in plants is closely related to changes in the levels of the assimilated molecules sugar, starch, and cellulose (Reid, 2019). Out of these molecules, sucrose and starch metabolism are interconnected and are responsible for plant growth and secondary metabolism. This regulation of genes involved in metabolism not only promotes root expansion but also influences the secondary metabolites in medicinal plants.

Important enzymes involved in sucrose metabolism are UDP-glucose pyrophosphorylase (UGPase), phosphoglucomutase (PGM), and sucrose synthase (SUS) (Duan et al., 2025; Hafeez et al., 2025; Zhang W et al., 2025). Genome-wide comparative analysis has uncovered a remarkable expansion of gene families contributing to sucrose metabolism. This includes the existence of 16 *INVERTASE (INV)* genes, 9 *SUCROSE PHOSPHATE SYNTHASE (SPS)* genes, and 12 *SUS* genes in medicinal plants. This genetic diversity has been achieved through tissue-specific gene expression and regulation of stress responses (Yuan et al., 2024). INV irreversibly hydrolyses sucrose into glucose and fructose, playing key roles in carbon allocation and stress responses in plants. *INV* genes comprise several subtypes: *CELL WALL INVERTASE* (*CWINV*) regulates sucrose unloading from the phloem to sink tissues, *VACUOLAR INVERTASE* (*VINV*) controls sucrose storage and degradation in vacuoles, and *ACID/NEUTRAL INVERTASE* (*A/N-INV*) functions in the cytoplasm or mitochondria, contributing to abiotic stress responses (Dahro et al., 2022; Ren et al., 2022; Zaman et al., 2025). In *Arabidopsis thaliana*, Sucrose-phosphate synthase (SPS) catalyzes the formation of sucrose-6-phosphate from UDP-glucose and fructose-6-phosphate, whereas SUS reversibly cleaves sucrose into UDP-glucose and fructose. The UDP-glucose generated by SUS serves as a key precursor for cellulose biosynthesis, thereby supporting cell wall formation and energy metabolism (Wang et al., 2022). The starch metabolism pathway is further modulated by several key enzymes: starch synthase (SS), starch phosphorylase (SP), and ADP-glucose pyrophosphorylase (AGPase) (Tetlow, 2011). Changes in the activity and expression levels of key enzymes influence sucrose and starch metabolism and allocation, ultimately affecting biomass accumulation and secondary metabolite production in the roots. Sucrose metabolism is active during development. Different enzymes have different levels of regulation. AGPase plays a key role in amylose and starch biosynthesis. Soluble starch synthase (SSS) promotes the formation of branched-chain starch (Ying et al., 2025). In *Pueraria lobata* tubers, the expression of *SUS* and *INV* remains high from transplantation through the late expansion stage. Before mid-expansion, *SPS* genes are strongly upregulated, while genes encoding granule-bound starch synthase (GBSS), starch branching enzyme (SBE), SSS, and isoamylase (ISA) promote starch biosynthesis. During the later stages, GBSS, SBE, and ISA remain predominantly upregulated, indicating stage-specific regulation of sucrose–starch partitioning (Xufeng et al., 2023).

In addition to their roles in structural development, the molecular processes underlying root enlargement are closely associated with the accumulation of bioactive compounds, which are key determinants of medicinal quality. Increasing evidence indicates that regulatory processes such as carbohydrate metabolism, hormonal signaling, and transcriptional regulation not only drive root thickening but also coordinate secondary metabolite biosynthesis.

During root thickening in *Panax ginseng*, secondary metabolites such as ginsenosides, polysaccharides, and phenolic compounds accumulate as major bioactive constituents. Their biosynthesis is closely associated with developmental stage, cell-type specificity, stress responses, and interactions with soil microorganisms (Cui et al., 2024; Zhan et al., 2025). In *Flemingia macrophylla* (Willd.) Merr., high levels of isoflavonoids, such as genistein and genistin, accumulate in lateral roots during the thickening process (Tan et al., 2025). Similarly, in *Angelica sinensis*, secondary metabolite biosynthesis is strongly influenced by soil microbial interactions, affecting the production of phenylpropanoids and polyketides, with lateral roots and inflorescences serving as key biosynthetic sites (Xu et al., 2025).

These findings highlight that root expansion represents a critical developmental stage during which molecular regulatory networks coordinate both structural growth and the biosynthesis of pharmacologically active compounds.

### 4.4 Transcription factors regulating the development of root enlargement

Gene regulation is a fundamental physiological process that promotes plant development and growth, including root development. This physiological process is enabled by transcription factors (TFs), key proteins that bind to cis-acting regulatory elements in gene promoters. This enables the control of downstream target gene expression.

#### 4.4.1 Short root

As mentioned before, while the primary vascular cambium controls the initiation of root primordia during expansion, the secondary vascular cambium promotes root growth by stimulating cell multiplication and hypertrophy of existing root cells. This transcription factor activity may help control medicinal plant root development by reducing cell proliferation and cellular expansion. A plant-specific transcription factor designated SHR, a member of the GRAS family of transcription factors, shows little similarity to other GRAS transcription factors with known activities; it conserves only the PFYRE and VHIID domains (Neves et al., 2023). This marked divergence of the SHR transcription factor places SHR at a distinct branch of the GRAS genetic tree. In *Arabidopsis thaliana*, SHR activity was found to be requisite for the initial development of the meristematic apparatus in plant roots (Hao and Cui, 2012). SHR and SCR synergistically maintain meristem activity by suppressing the CTK response (Fu et al., 2021). In *Arabidopsis thaliana*, SHR participates in the maturation of root meristem tissue by promoting periclinal division, a process jointly regulated by brassinosteroid (BR) and reactive oxygen species (H_2_O_2_) signaling. The BR-activated transcription factor BZR1 directly binds to the *SHR* promoter to induce its expression and finely regulate periclinal division through the BZR1-SHR transcriptional module (Tian et al., 2022). Additionally, in *Arabidopsis thaliana*, SHR influences auxin distribution by regulating the expression of the auxin transporter *PIN1*, thereby affecting meristem size and the expression of stem cell regulatory factors such as *PLT1/2* (You et al., 2024). SHR inhibits BR signaling in mature root zones, thereby activating downstream lignin-synthesis-related genes and initiating cell wall lignification, marking the developmental transition of meristematic tissue to elongation zones in *Arabidopsis thaliana* (Li et al., 2022). The primary root length of *shr* and *scr* mutants is significantly reduced. The deficiency of *miR165A* and *miR166B* expression caused by the *shr* mutation led to a lack of primary xylem tissue (Hao and Cui, 2012). The trans-factor participates directly in cell division and tissue differentiation in root tissues; hence, it was found to affect plant root development in *Arabidopsis thaliana*.

SHR and SCR together constitute the primary regulatory module governing the formation of the stroma’s organizational pattern in *Arabidopsis thaliana*. By finely regulating the spatiotemporal expression of the cell cycle gene *CYCLIND6;1* (*CYCD6;1)*, they control the formative division of the initial cells of the endoderm and ectoderm (CEI/CEID), as well as the development of the mesoderm (Xie et al., 2023). SCR acts as a transcription factor that plays a vital role in root development; hence, it is expressed in CEI cells and the endodermal layers (Liu et al., 2024). In CEI D cells, SHR stimulates *SCR* gene transcription; thereafter, SCR sustains a higher level of transcription with the help of positive feedback regulation. SHR and SCR simultaneously induce transcription of the *CYCD6;1* and other cyclins which eventually activate CEI and CEID cell division (Pauluzzi et al., 2012). Notably, SCR also promotes the expression of *SHR INTERACTING EMBRYONIC LETHAL (SIEL)*, which further induces the transport of SHR from the stele to the endodermis and CEI cells (Hirano et al., 2017). Functionally, SHR functions as an upstream transcriptional regulator of *SCR* (Dong et al., 2021), where the interaction between SHR and SCR not only strengthens SHR signaling but also inhibits the promotion of excessive periclinal cell division of the basal tissue. Through this interaction, SHR-SCR signaling further sustains high SHR levels specifically in the endodermis, thereby modulating organ thickening and root growth (Koizumi et al., 2012).

#### 4.4.2 Auxin/Indole-3-acetic acid

Auxin, a plant growth hormone, participates in a wide range of plant developmental processes and regulates fundamental cellular activities, including cell division, cell growth, and cell differentiation (Ding et al., 2021; Templalexis et al., 2025). In the model plant *Arabidopsis thaliana*, auxin induction and depletion induce gene programming, a biological response mediated by AUX/IAA transcriptional regulators. Auxin-mediated transcriptional regulation relies totally on the activity of AUX/IAA proteins (Lavy and Estelle, 2016). *AUX/IAA* genes belong to a plant-exclusive family that encodes transcription regulators responsible for modulating the AUX-responsive transcription of genes involved in AUX-regulated plant development processes in a tissue and developmentally specific way (Chandler, 2016). AUX/IAA proteins regulate auxin-responsive transcription to control the development and growth of plant roots by modulating hormonal levels during plant development. Auxins respond to plant cell signaling to regulate root development by relieving the repression of AUX/IAA proteins on the transcriptional regulators ARFs. This relieving response increases the transcription of genes responsible for plant roots, thereby promoting plant cell division and expansion, and hence plant root system development and expansion (Cavalleri et al., 2024).

The function of AUX/IAA proteins in regulating root development was studied in ginseng, where 84 *AUX/IAA* genes have been identified in the genome. Transcript analysis by RNA-seq indicated that *AUX/IAA* genes have tissue-specific expression. In *Panax ginseng*, the *AUX/IAA* genes *PgIAA02* and *PgIAA36* are highly expressed in fibrous and lateral roots. Functional analysis revealed that overexpression of *PgIAA02* strongly inhibited lateral root growth. In addition, PgIAA02 interacts with PgARF22 and PgARF36 (AUX response factors), specifically localizing to nuclei to regulate root development. All the observations indicate that PgIAA02 suppresses the expansion of ginseng roots by binding to ARF proteins (Wang Y et al., 2024).

AUX/IAA proteins play a vital role in the development of plant roots. In *Arabidopsis thaliana*, *AtIAA16* and *AtIAA20* are implicated in primary root development, while *AtIAA3*, *AtIAA8*, and *AtIAA12* function during lateral root development by mutation or gain-of-function (Guseman et al., 2015). Auxin also regulates the initiation of root meristem organization via MP/ARF5-dependent transcriptional changes, a process that requires the PB1 domain of BDL/IAA12 and the C-terminal domain of MP/ARF5 (Cavalleri et al., 2024). Until then, *AUX/IAA* genes have been identified and functionally analyzed in several plant species, ranging from model plants to crops and cash crops (Paul et al., 2016; Liu et al., 2017).

#### 4.4.3 NAM/ATAF/CUC

NAC transcription factors are plant-specific proteins that share common characteristics with a high level of similarity due to the presence of an N-terminal DNA-binding domain (~150 amino acids), followed by a C-terminal transcriptional regulatory region (TRR). This N-terminal domain can be further divided into five subdomains (A-E) (Singh et al., 2021). Both the N-terminal and C-terminal domains are responsible for target gene interactions that help NAC proteins serve multiple roles. NAC transcription factors participate in several plant cell processes, including secondary cell wall regulation (Xu et al., 2021), leaf senescence (Lee and Masclaux-Daubresse, 2021), secondary metabolism of bioactive compounds (Meraj et al., 2020), stress responses (Dudhate et al., 2021), and others for fruits to ripen, meristems to develop at tips, and floral organ acquisition (Massafra et al., 2025).

Regarding plant root expansion in *Ipomoea batatas*, analysis of the dynamic network biomarker (DNB) of the transcriptome during storage root development highlighted the NAC transcription factor IbNAC083 as a key regulator of root initiation within the DNB-related network. Functional analysis indicated that *IbNAC083* and its related differentially expressed genes acted as modulators of key biochemical pathways for lignin, flavonol, and starch biosynthesis, thereby inducing the transition to expanded roots (He et al., 2021). In *Arabidopsis thaliana*, NAC1 inhibits cell division of the meristematic cells of the root endodermis by directly inhibiting the cell cycle regulator *CYCD6;1.* This repression is mediated by the recruitment of the co-repressor TOPLESS (TPL) to form an NAC1-TPL complex that interacts with the *CYCD6;1* transcriptional start site. NAC1 also physically interacts with the transcription factors SCR and SHR to co-repress uncontrolled cell division in endodermal cells, thereby promoting proper patterning of the root meristem. NAC and SHR display an antagonistic regulation where NAC1-TPL complex repression of *CYCD6;1* transcription is SCR-dependent; NAC and SHR also antagonize each other to modulate the *CYCD6;1* expression for accurate control of cortical cell division (Xie et al., 2023). NAC1 can also activate peptide hormone genes such as *AtCEP1/2* and indirectly regulate root morphology by preventing root hair extension (Rodríguez-García et al., 2023). Under reduced nitrate (NO_3−_) concentrations, NAC075 (LONR1) moves from the xylem to the inner cortex of the roots due to CIPK1-mediated phosphorylation. After transport, NAC075 coordinates downstream targets such as *WRKY53* to transform the roots in response to nutrient stress (Xiao et al., 2022). In ginseng, a total of 197 *NAC* genes have been found. Phylogenetic and expression analyses indicated that 23 genes were absent from the roots. They can presumably be involved elsewhere, for instance, in seeds or lateral roots. Others can be involved in stress responses and hormonal regulation.

#### 4.4.4 PLETHORA

PLETHORA (PLT) transcription factor family members are recognized to play fundamental roles in regulating root organogenesis, primarily characterized in the model *Arabidopsis thaliana*, *plt* mutants with deficiencies in several *PLT* genes display severe meristem disorganization at the root tip, including disruption of the stem cell niche. While the level of *WOX5* gene expression is not drastically reduced in *plt1/plt2* double mutants,in *Arabidopsis thaliana*, *PLT1* ectopic expression causes the expansion of the *WOX5* expression territory to cortical endodermal initial daughter cells, indicating that *PLT* genes control the boundaries of the stem cell niches by controlling *WOX5* expression patterns (Deng et al., 2025). *PLT* overexpression during embryonic development induces ectopic root meristems, while partial PLT levels induce proliferation of transit-amplitude cells with reduced cell elongation, demonstrating a PLT dose effect on meristem division at the root tip in *Arabidopsis thaliana*. Analysis of transcriptional regulation provides further evidence that *PLT* genes are cell-fate regulators of plant organogenesis by promoting cell division while repressing cellular differentiation. The coordinated function of PLTs in root meristematic tissue relies on protein gradients across the meristem. In stem cells with high PLT levels, PLT activates *miR396*, which degrades mRNAs encoding growth regulators (GRFs), thereby restricting *PLT* expression in transit-amplifying cells. Intercellular mobility and dilution by cell division establish these gradients, which are interpreted through dose-dependent responses along the longitudinal axis of the meristem. PLT protein stability is largely dependent on ROOT GROWTH FACTOR (RGF)/GLUTININ (GLV) peptide signaling. Exogenous RGF/GLV application mimics *PLT* overexpression by expanding apical meristematic tissues, whereas higher-order mutants defective in RGF/GLV or its receptor show severe meristem defects and reduced PLT protein levels. Lateral root formation requires auxin-dependent activation of transcription factors *ARF7*, *ARF19*, and *LATERAL ORGAN BOUNDARIES DOMAIN16* (*LBD16).* ARF7 and ARF19, core components of the auxin signaling pathway, regulate downstream targets such as *LBD16* and *LBD18*, thereby driving lateral root initiation (Caselli et al., 2025). Under salt stress, ARF7/ARF19 binds the *SOS1* coding region to induce expression, while the ubiquitin ligase CHYR1 negatively regulates this process by promoting ARF7/ARF19 degradation (Lu et al., 2025). Indeterminate root formation depends on auxin-mediated activation of *LBD16* and *WOX11*, as reported in model and crop species including *Arabidopsis thaliana* and *Oryza sativa* (Stitz et al., 2023). Radial expansion and lateral root induction are closely linked to shoot-derived signals. Auxin functions as a mobile signaling molecule coordinating above- and belowground development, although the transcription factor HY5 is also proposed as a mobile mediator of shoot-root communication in light-dependent regulation (Kircher and Schopfer, 2023). Other transcription factors, such as SRF1, the MADS-box protein SRD1, and auxin-responsive ARFs, have been intensively studied. SRF1 modulates carbohydrate metabolism in nodules by negatively regulating vesicular transport genes, while SRD1 promotes nodule formation by inducing proliferation in the formation layer and epithelium (Noh et al., 2013).

#### 4.4.5 Other transcription factors

DELLA proteins are growth-repressing transcription factors that play central roles in regulating plant growth, development, and root expansion. As key regulators in phytohormone signaling, DELLA proteins integrate multiple cues to modulate cellular processes. In nodule development and root growth, the GA signaling pathway is closely associated with organ expansion. The core GA pathway repressor, DELLA, inhibits GA-mediated growth by orchestrating transcriptional reprogramming, thereby restraining cell proliferation and elongation (Sun, 2011).

**Figure 3:**
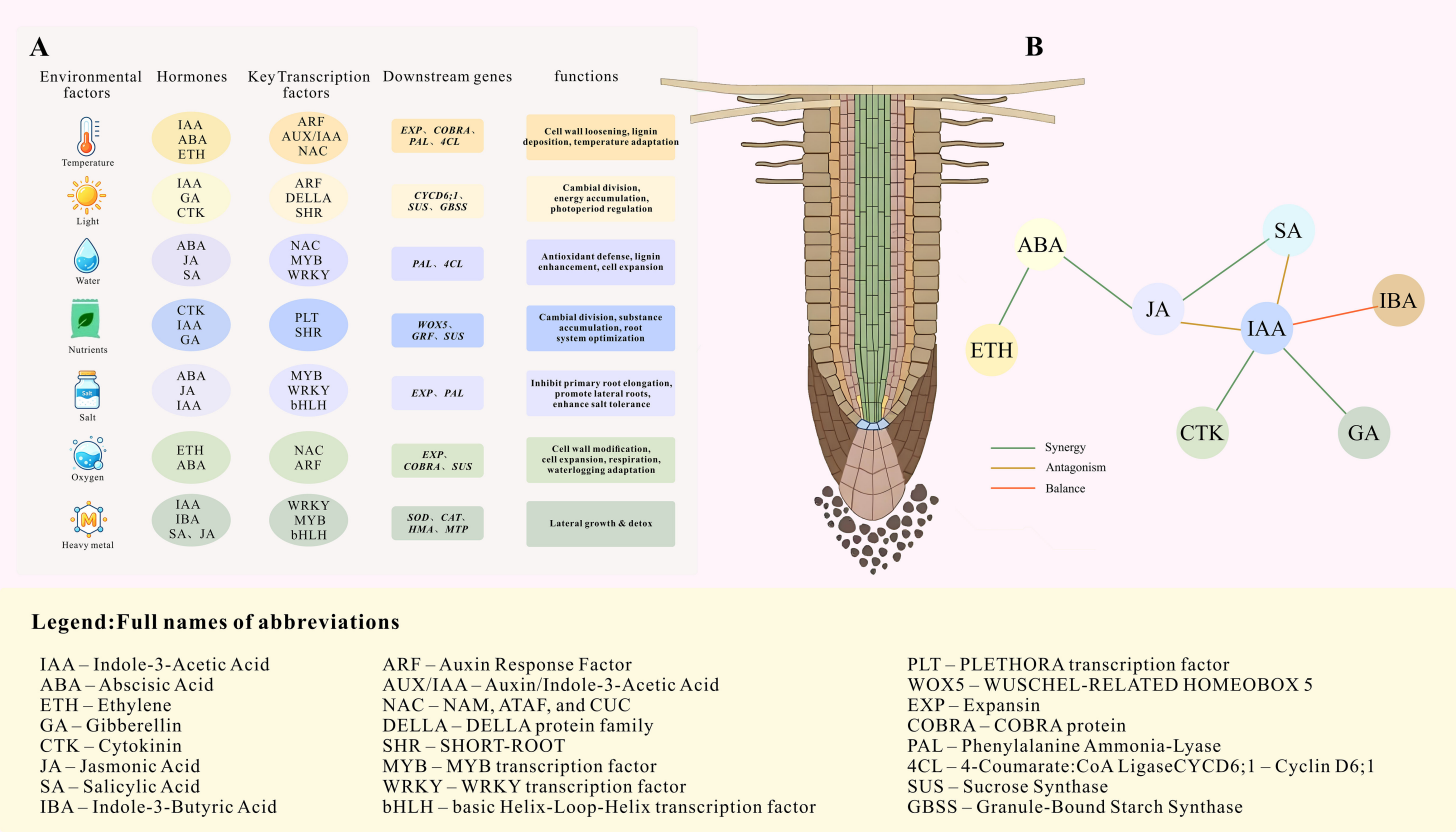
Integrated regulatory network of environmental cues, phytohormones, and transcription factors controlling root expansion and thickening. **(A)** Environmental factors such as temperature, light, water availability, nutrient status, salinity, hypoxia, and heavy metal stress regulate root expansion and thickening through coordinated phytohormone signaling pathways. These hormonal signals activate key transcription factors, such as ARF, AUX/IAA, NAC, MYB, WRKY, PLT, and SHR, which subsequently regulate downstream genes involved in cell wall remodeling, lignin biosynthesis, cell division, carbohydrate metabolism, antioxidant defense, and root architectural development. These regulatory cascades ultimately influence root growth, cambial activity, stress adaptation, and resource acquisition. **(B)** Schematic representation of hormone crosstalk during root development and expansion, illustrating the regulatory relationships described above. IAA serves as a central regulatory hub interacting with ABA, ETH, JA, SA, CTK, GA, and IBA. Green lines indicate synergistic interactions, yellow lines indicate antagonistic interactions, and red lines indicate balancing effects. The root-tip diagram highlights the central role of hormone-mediated regulation in root growth and developmental responses.

Other families of transcription factors (TFs), such as WRKY and MYB, are also involved in the expansion of medicinal plant roots by regulating downstream target genes that control cellular differentiation, proliferation, and the biosynthesis of active ingredients. Because species differences exist in the transcription factor-mediated regulatory system of medicinal plant development and metabolism, a detailed analysis of the molecular mechanism of root expansion and its transcription factor family members for each species can be a key strategy for understanding developmental regulation and improving the utilization of medicinal plant resources. An integrated view of how environmental cues, phytohormones, and transcription factors coordinately regulate root expansion and thickening is presented in **Figure 3**.

### 4.5 Organ-specific differentiation and evolutionary implications of root expansion

Specialized and intriguing examples of plant organogenesis include localized hypertrophy of subterranean plant organs, such as the thickening of the primary root of *Daucus carota*, in which the primary root becomes bulky with a slender lateral root system. This specificity of plant organogenesis appears far from random and instead requires careful integration of diverse factors, including physiological requirements, gene expression, and evolution (Macko-Podgórni et al., 2020). This chapter presents a consolidated view of diverse hypotheses that could account for the specific expansion of plant roots across different plant organs.

#### 4.5.1 Functional differentiation and resource allocation

Functionally, primary and lateral roots differ in their specific roles in a plant’s life cycle.

A primary root serves as the main storage organ that accumulates starches, proteins, and assimilates for regeneration or plant reproduction. This can be evidenced by the transient accumulation of starches and proteins in rapeseed roots (James et al., 2025). On the other hand, lateral roots serve mainly for anchorage and nutrient absorption. This kind of division of labor means that there may be a trade-off between plant evolution toward increased allocation of certain plant nutrients to storage structures, since becoming thicker consumes substantial plant energy. This means that lateral roots remain thin, with a high surface-area-to-volume ratio, thereby enhancing absorption at a lower cost to the plant (Fan et al., 2023; Zanetti et al., 2024).

#### 4.5.2 Molecular regulation of developmental specificity

Functional differentiation relies on molecular diversity. Evidence from Raphanus sativus (radish) demonstrates that specific transcription factors or signaling cascades exhibit spatiotemporal patterns of gene expression in thickened areas. *WOX* genes (e.g., *RsWOX13*), which are central regulators of cambial activity and secondary growth, induce cell division and radial expansion of the primary roots of plants (Plunkert et al., 2024; Zhang X et al., 2025). The absence of activating peptide (e.g., *CLEs*) genes can impede the initiation of this signaling system in lateral roots and hence their ability to thicken. A mutation that inserts a C-terminal tag into the gene for LBD transcription factor *RsLBD3* increases its transcriptional activity; hence, lateral root thickening can only occur due to the increased transcriptional activity of this gene. Otherwise, its lower transcriptional activity limits root extension and biomass accumulation (Dong et al., 2025).

#### 4.5.3 Hormonal microenvironment and local growth signaling

Organ-level specificity may also be modulated by local hormone gradients. A local hormone microenvironment hypothesis can be stated as follows: In the thickening zone of the primary root, specific local hormone environments with higher concentrations of auxins or brassinosteroids (BRs) may develop due to local biosynthesis rather than transport. Hormones influence local cells to initiate programs for thickening (Jia et al., 2021; Vukašinović et al., 2021), whereas in lateral roots, hormonal dynamics are primarily involved in gravitropic responses and growth for environmental adaptation rather than storage.

#### 4.5.4 Growth-defense balance and environmental modulation

On one hand, the developmental trajectory of storage roots should strike a balance between growth and defense reactions. *Rehmannia glutinosa*, for example, experiences continuous cropping stress that promotes excessive cell lignification to the point that regular tuber growth is impeded (Gu et al., 2021). Notably, this indicates that a target developmental timing range with a perfect balance between stress and metabolism is needed for root thickening to occur; however, lateral roots that serve as the first defense in the rhizosphere may prioritize defense hardening.

#### 4.5.5 Phylogenetic constraints and evolutionary significance of storage root formation

Notably, storage root formation is not a universal trait among higher plants but represents a lineage-specific evolutionary strategy. The capacity for radial expansion requires pre-existing anatomical features, including persistent or reactivatable vascular cambium, high proportions of parenchymatous storage tissues, and delayed lignification. In addition, an integrated regulatory network involving hormone gradients, transcription factor modules, and carbon allocation signaling must be established in a coordinated manner (Eserman et al., 2018).

Therefore, storage root enlargement occurs only in plant lineages that possess both the anatomical competence and the molecular regulatory framework necessary for sustained secondary growth. From an evolutionary perspective, the emergence of storage roots reflects adaptive selection under seasonal climates, drought stress, or nutrient fluctuation, where enhanced underground carbon storage confers survival and reproductive advantages. In contrast, species lacking these structural and regulatory prerequisites do not exhibit storage root hypertrophy (Vanderschuren and Agusti, 2022).

## 5 Concluding remarks

Plants that belong to the medicinal plant type, where roots are the main pharmacologically active organ, take a big share of the plant species of China’s medicinal plant bank. Therefore, tuberous root development directly affects drug activities and socio-economic benefits. Therefore, studying tuberous root development and expansion in medicinal plant species is of great scientific and applied value. However, existing scientific studies have been focused on the effects of endogenous hormones on tuberous root development and expansion. Plant hormones are key regulators of plant growth and development; therefore, exogenous applications of plant growth regulators have been explored for manipulating tuberous root development (Utsumi et al., 2022; Jia et al., 2025). But scientific studies on tuberous root development and expansion of medicinal plants have been inadequate. Very few medicinal plant species have been explored; hence, existing scientific studies have focused on the effects of endogenous hormones and exogenous plant growth regulators on tuberous root development. At the molecular level, few scientific studies exist; therefore, understanding tuberous root expansion in medicinal plant species remains inadequate.

This study aims to elucidate the molecular mechanism underlying root extension in medicinal plants. Based on the discovery of key factors influencing root extension, we identified several genes and transcription factors involved in sucrose and starch metabolism, lignification, and hormonal regulation. These genes and transcription factors are vital to cell division, cell differentiation, and root extension. At the same time, some important genes and regulatory pathways involved in the accumulation of active ingredients for root extension have been identified, laying a theoretical basis for further study of active compound accumulation in medicinal plants. These findings reveal the mechanistic basis for the formation of authenticity, providing empirical evidence for the scientific definition of authentic medicinal materials. However, some problems exist despite the current progress. Firstly, the specific functions and regulation paths for some important genes to influence the extension of roots have not been thoroughly clarified and require further clarification. Secondly, the biochemical pathways and gene regulation underlying active compound accumulation have not been fully elucidated and warrant further study. Further study should emphasize the verification of key genes, multi-omics analyses of root extension and active compound biosynthesis in medicinal plants, and interdisciplinary research integrating plant physiology, gene analysis in medicinal plants, and pharmacology. This study can give theoretical support for plant breeding and the utilization of medicinal plants.

In conclusion, this review can serve as a reference for understanding the molecular mechanisms underlying root expansion and the accumulation of bioactive compounds in medicinal plants. At the same time, this review highlights some of the remaining challenges and provides direction for future research to explore the complex control system underlying root expansion and bioactive compound accumulation. With the increasing availability of genomic and transcriptomic resources for medicinal plants, future comparative and functional genomics studies will further improve our understanding of the regulatory mechanisms underlying root enlargement and the accumulation of bioactive compounds.
